# Lichenoid esophagitis presenting as fatal upper gastrointestinal bleeding in a 52 year-old woman: a case diagnosed by autopsy

**DOI:** 10.1186/s12876-017-0647-0

**Published:** 2017-08-08

**Authors:** Andrew Mitchell, Tony Petrella

**Affiliations:** Department of Anatomical Pathology and Cytology, Maisonneuve-Rosemont Hospital, 5415 Boulevard de L’Assomption, Montreal, Quebec H1T 2M4 Canada

**Keywords:** Lichenoid, Esophagitis, Lichen planus, Autopsy, Case report

## Abstract

**Background:**

“Lichenoid esophagitis” is a descriptive term for a lichenoid pattern of inflammation in the esophagus for which a precise histologic diagnosis cannot be established. The differential diagnosis includes lichen planus, a drug-related reaction, and viral infection. Lichenoid esophagitis causing death has not been reported previously. We describe a case, diagnosed by autopsy, of lichenoid esophagitis in which massive bleeding from generalized epithelial sloughing and a large longitudinal ulcer proved fatal.

**Case presentation:**

A 52 year-old diabetic woman collapsed at her home in front of an acquaintance. “Bloody vomit” was noted. Despite resuscitation efforts, the patient died. A complete autopsy was performed. The middle portion of the esophagus showed a 9 cm longitudinal ulcer situated 12 cm from the esophago-gastric junction. Microscopic examination showed complete sloughing of the esophageal epithelium with a striking subepithelial lichenoid lymphocytic infiltrate extending into the muscularis mucosae. The findings were considered compatible with lichenoid esophagitis. Laboratory studies also showed the presence of diabetic ketoacidosis.

**Conclusions:**

Lichenoid esophagitis is an appropriate diagnostic term when clinical, histologic and laboratory findings do not allow for specific categorization of lichenoid inflammation in the esophagus. As illustrated here for the first time, lichenoid esophagitis may cause ulceration and mucosal sloughing severe enough to result in massive upper gastrointestinal bleeding and death. Translating these autopsy findings to the clinical setting, it is possible that the endoscopic finding of a longitudinal mid-esophageal ulcer in the presence of proximal stricture may be indicative of underlying lichenoid esophagitis.

## Background

“Lichenoid esophagitis” is a descriptive term for a lichenoid pattern of inflammation in the esophagus for which a precise histologic diagnosis cannot be established [1]. The differential diagnosis includes lichen planus (LP), a drug-related reaction, and viral infection [1]. Lichenoid esophagitis causing death has not been reported previously. We describe a case, diagnosed by autopsy, of lichenoid esophagitis in which massive bleeding from generalized epithelial sloughing and a large longitudinal ulcer proved fatal.

## Case presentation

A 52 year-old woman collapsed at her home in front of an acquaintance. “Bloody vomit” was noted. An ambulance was called and cardiopulmonary resuscitation (CPR) was begun by paramedics. The patient was transported to hospital, but she remained in cardiorespiratory arrest despite ongoing CPR. Resuscitation efforts were finally stopped.

It was later learned that several days prior to her death she had complained of unspecified upper digestive symptoms, but had steadfastly refused to seek medical advice. The medical history included bipolar disorder for which she was apparently compliant in taking her medications. She also smoked and had type II diabetes. Her medications were the following: quetiapine, lorazepam, baclofen, glickazide, metformin, and esomeprazole.

The coroner was notified and an autopsy was requested.

### Autopsy and laboratory results

**Fig. 1:**
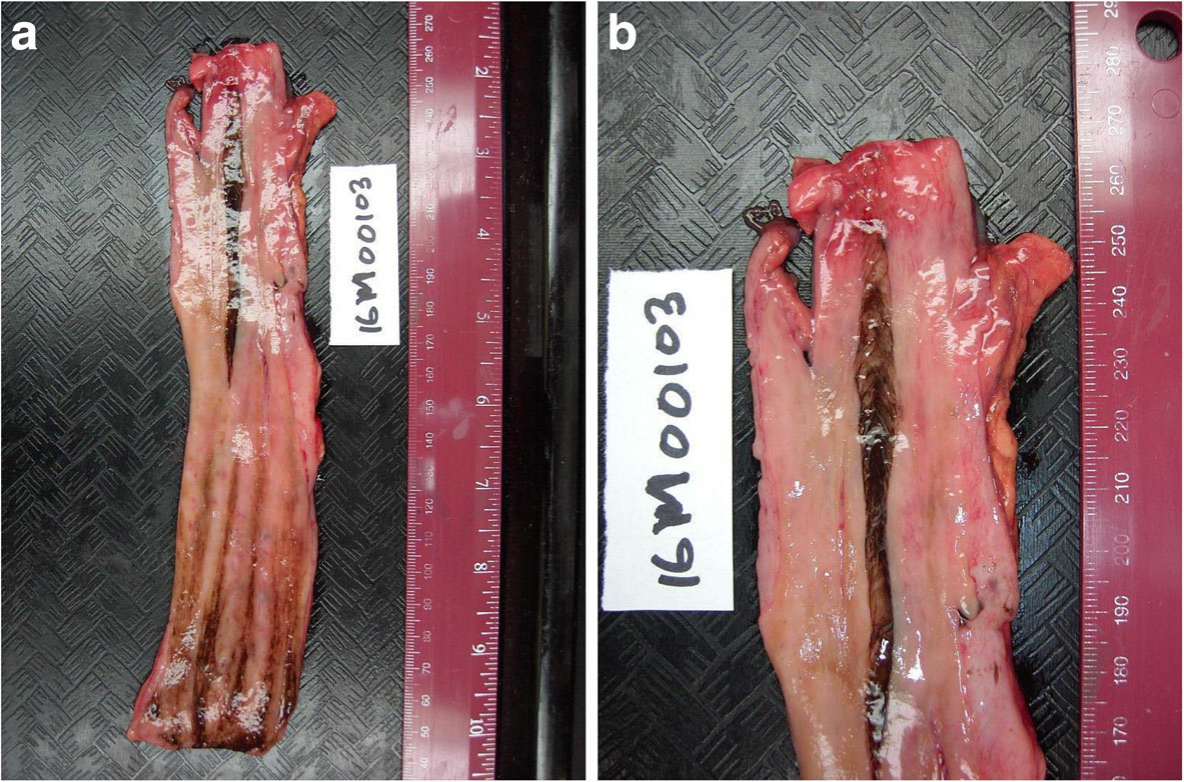
Macroscopic images of the esophagus. **a** The mid-esophagus shows a 9 cm ulcer with a brown base. The esophago-gastric junction (bottom) has brownish streaks without ulceration. The rest of the esophagus is unremarkable despite the marked inflammation and complete absence of epithelium. **b** A closer view of the ulcer

A complete autopsy was performed. The pertinent anatomic findings were confined to the gastrointestinal tract. The middle portion of the esophagus showed a prominent brownish 9 cm longitudinal ulcer, with a maximal width of 0.5 cm, situated 12 cm from the esophago-gastric junction (Fig. 1). The esophago-gastric junction itself showed brownish steaks without ulceration, induration or a mass lesion. The stomach contained approximately 750 ml of dark blood; the small bowel and colon contained dark, tarry blood. There was no identifiable mucosal lesion beyond the esophagus.

**Fig. 2:**
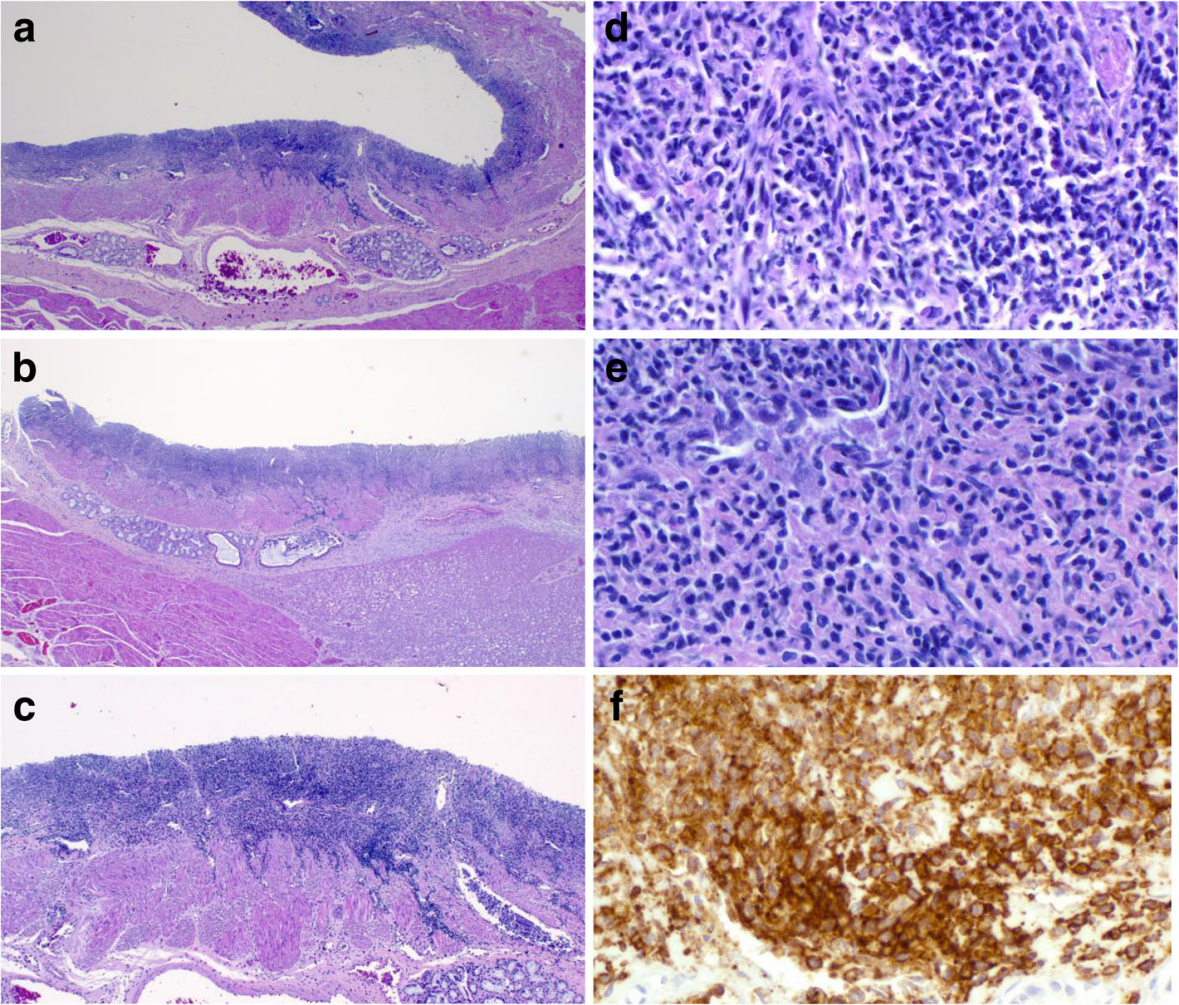
Microscopic images of the esophagus. **a, b** Low power images of the esophageal wall. The epithelium has entirely sloughed off. A dense lichenoid lymphocytic infiltrate involves the lamina propria and muscularis mucosae. Esophageal glands in the submucosa are uninvolved. **c** Medium power image of the lichenoid infiltrate showing extension into the muscularis mucosae. The epithelium has entirely sloughed off. **d**, **e** Medium-high power images of the lymphoid infiltrate. The twisted appearance of many of the lymphocyte nuclei is typical of T-cells and does not imply atypia. **f** Medium-high power view of the lymphocytes showing uniformly strong positivity with the T-cell marker CD43

Microscopic examination of numerous sections (>30) from all levels of the esophagus, including the ulcer, showed complete sloughing of the epithelium with a striking subepithelial lichenoid lymphocytic infiltrate extending into the muscularis mucosae (Fig. 2, a-e). Sections of the esophago-gastric junction showed no evidence of varices. Fungal elements and viral inclusions were absent; Periodic acid-Schiff staining for identification of fungi and immunohistochemical staining for Cytomegalovirus and Herpes simplex viruses I and II were negative.

An extensive immunohistochemical study was performed in order to characterize the immunophenotype of the lymphocytic infiltration. The following antibodies were used: CD20, CD2, CD3, CD4, CD5, CD7, CD8 and CD43. Unfortunately, marked post-mortem autolysis rendered interpretation difficult. Only CD43 staining was well preserved (Fig. 2f), showing diffuse positivity, thus demonstrating that the lymphocytic infiltrate was essentially composed of T-cells. For the other antibodies the results were less reliable, although better antigen preservation of the deeper situated lymphocytes in the muscularis mucosae was noted. Here, a high CD8/CD4 ratio was found. CD2, CD5, CD7 and CD20 were negative, but, given the absence of positive controls for these markers, interpretation was essentially impossible.

A representative formalin-fixed, paraffin-embedded tissue block was submitted for evaluation of T-cell receptor gamma chain rearrangement by polymerase chain reaction. However, amplification was not possible for technical reasons.

Laboratory results were the following: lorazepam at a therapeutic blood level; tetrahydrocannabinol metabolites were also present. Ethanol was absent. Blood acetone: 10 mmol/L (normal: 0–0.34), ocular liquid glucose: 640 mg/dL, ocular liquid lactic acid: 230 mg/dL, and ocular liquid acetone: 12 mmol/L. The results were compatible with diabetic ketoacidosis (DKA).

The final autopsy diagnoses were: 1) fatal upper gastrointestinal bleeding due to lichenoid esophagitis with diffuse mucosal sloughing and a large ulcer of the mid-esophagus, and 2) diabetic ketoacidosis.

## Discussion and conclusions

To the best of our knowledge this is the first description of fatal hemorrhage due to esophagitis with a lichenoid inflammatory pattern. This is also the initial description of a macroscopically distinctive mid-esophageal ulcer in this entity.

“Lichenoid esophagitis” is the descriptive term proposed by Salaria et al. [1] for a lichenoid pattern of inflammation in the esophagitis for which a precise histologic diagnosis cannot be established. This may be due to inadequate or unavailable clinical information and/or laboratory data. The differential diagnosis includes LP, a drug-related reaction, and a viral infection [1]. These entities are discussed below in regard to the present case.

LP is the prototypical lesion of the esophagus characterized by lichenoid inflammation [1–5]. LP is a mucocutaneous inflammatory disorder, likely of autoimmune cause, that most commonly affects the skin and oral mucosa [5]. Skin lesions usually resolve within a year, whereas mucosal disease tends to be chronic [5]. Although esophageal involvement is considered rare, with approximately fifty reported cases as of 2013 [3], it is likely under-recognized [1]. Clinically, esophageal LP is characterized by dysphagia (with or without odynophagia) with endoscopic findings that include mucosal sloughing and stricture, small caliber of the esophagus, and superficial ulceration [1, 4]. Chronic stricture and squamous cell carcinoma are possible complications [4, 5].

There was no known history of LP in our case. Although a perfunctory external examination of the body prior to autopsy showed no obvious skin lesions, absence of skin lesions would not rule out LP as any skin lesions may have healed [5], and esophageal LP may be the first or only disease manifestation [4]. Given the complete mucosal sloughing, histologic evaluation of the epithelium was not possible. Thus, esophageal LP could not be confirmed by clinical evaluation or histologic criteria.

The diffuse mucosal sloughing also allowed only suboptimal assessment for viral inclusions. Inclusions were not found in the viable areas of the submucosa. As noted, immunohistochemical stains for Cytomegalovirus and Herpes simplex viruses I and II were negative.

The patient took multiple (six) prescribed medications. Salaria et al. found that 62% of patients with a lichenoid pattern of esophageal inflammation were taking more than three medications [1]. 29% of patients took a proton pump inhibitor; 32% took antihypertensive medications: angiotensin converting enzyme inhibitors, beta-blockers, calcium channel blockers, hydrochlotrothiazide, or a combination. A small number of patients in the study were receiving steroids and immune modulating medications for treatment of their esophageal disease. In the present case, as assessment of clinical and/or histologic response to withdrawal of medications was clearly not an option, it is impossible to establish if polypharmacy was responsible.

Thus, in light of the above, “lichenoid esophagitis” was considered the most appropriate term for the histologic findings.

The history of diabetes and the laboratory evidence of DKA should also be addressed, given the known association between acute esophageal necrosis (AEN), and diabetes [6–8]. AEN, also called “black esophagus” because of the endoscopic appearance of the necrotic epithelium, occurs in several critical care situations [6, 7]. Nearly 90% of patients present with upper gastrointestinal hemorrhage. There is a strong association with hyperglycemia; several cases associated with DKA have been reported. Necrotic mucosa is consistently found circumferentially at the esophago-gastric junction with varying degrees of proximal extension [6, 7]. Microscopically, there is necrosis of the mucosa and submucosa with denuded epithelium, necrotic debris and marked acute inflammation [8]. Proposed pathophysiologic mechanisms include mucosal ischemia, diminished mucosal defenses, and damage from gastric reflux [6]. Approximately 30% of patients die [6]. However, while upper gastrointestinal hemorrhage and laboratory evidence of DKA in this patient may initially suggest AEN, the pathologic findings eliminate this possibility: marked lichenoid inflammation, absence of acute inflammation, and the presence of a prominent ulcer in the mid-esophagus are not characteristic of AEN.

Finally, it is remarkable that this patient had, simultaneously, acute manifestations of two chronic conditions: severe esophageal ulceration secondary to lichenoid esophagitis and DKA complicating diabetes. Although we cannot be certain, dysphagia and bleeding from severe esophageal disease may have led to poor blood glucose control and subsequent DKA. One may also speculate as to the role of diabetes as a causative factor for lichenoid esophagitis. However, as ours is a description of a single case, further reports of this association would be needed to establish such a link.

In conclusion, lichenoid esophagitis is an appropriate diagnostic term when clinical, histologic and laboratory findings do not allow for specific categorization of lichenoid inflammation in the esophagus. This case illustrates that lichenoid esophagitis may cause ulceration and mucosal sloughing severe enough to result in massive upper gastrointestinal bleeding and death. Translating these autopsy findings to the clinical setting, it is possible that the endoscopic finding of a longitudinal mid-esophageal ulcer, especially in the presence of proximal stricture, may be indicative of underlying lichenoid esophagitis. However, further case descriptions will be necessary to provide confirmation.

## Abbreviations

AEN: acute esophageal necrosis
CPR: cardiopulmonary resuscitation
DKA: diabetic ketoacidosis
LP: lichen planus
